# Clinical Features of Coxsackievirus A4, B3 and B4 Infections in Children

**DOI:** 10.1371/journal.pone.0087391

**Published:** 2014-02-04

**Authors:** Chia-Jie Lee, Yhu-Chering Huang, Shuan Yang, Kuo-Chien Tsao, Chih-Jung Chen, Yu-Chia Hsieh, Cheng-Hsun Chiu, Tzou-Yien Lin

**Affiliations:** 1 Department of Pediatrics, Chang Gung Memorial Hospital at Linkou, Kweishan, Taoyuan, Taiwan; 2 Chang Gung University College of Medicine, Kweishan, Taoyuan, Taiwan; 3 Department of Laboratory Medicine, Chang Gung Memorial Hospital at Linkou, Kweishan, Taoyuan, Taiwan; Duke-National University of Singapore Graduate Medical School, Singapore

## Abstract

**Background:**

Clinical features of coxsackievirus A4 (CA4), B3 (CB3) and B4 (CB4) infections in children have not been comprehensively described.

**Methods/Principal Findings:**

From January 2004 to June 2012, a total of 386 children with culture-proven CA4, CB3 and CB4 infections treated at Chang Gung Memorial Hospital, including 296 inpatients (CA4, 103; CB3, 131; CB4, 62) and 90 outpatients (CA4, 55; CB3, 14; CB4, 21), were included. From outpatients, only demographics were extracted and from inpatients, detailed clinical and laboratory data were collected retrospectively. The mean age was 32.1±30.2 months; male to female ratio was 1.3∶1. Children with CB3 infection were youngest (76.6% <3 years of age), and had a highest hospitalization rate (90.3%) and a longest duration of hospitalization (mean ± SD, 7.5±6.2 days). Herpangina (74.8%) was the most common presentation for children with CA4 infection, aseptic meningitis (26.7%) and young infant with fever (23.7%) for those with CB3 infection, and herpangina (32.3%) and tonsillitis/pharyngitis (27.4%) for children with CB4 infection. Almost all the inpatients had fever (97.6%). Twelve out of thirteen (92.3%) children with complications and ten of 11 children with long-term sequelae had CB3 infections. Two fatal cases were noted, one due to myocarditis with CA4 infection and CB3 were detected from the other case which had hepatic necrosis with coagulopathy. The remaining 285 children (96.3%) recovered uneventfully.

**Conclusion:**

CA4, CB3 and CB4 infections in children had different clinical disease spectrums and involved different age groups. Though rare, severe diseases may occur, particularly caused by CB3.

## Introduction

Enterovirus are common pathogens in pediatric infectious disease and traditionally classified into poliovirus, coxsackievirus A (CA), coxsackievirus B (CB), echovirus, and the enteroviruses named with numbers such as enterovirus 68 to 71 [1]. International Committee on Taxonomy of Viruses adopted a new classification of human enteroviruses (HEVs) in 2000 and then HEVs are subgrouped into five species: poliovirus, HEV-A, HEV-B, HEV-C, and HEV-D; according to the similarities in their viral structure protein (VP) genes [2].

Non-polio enteroviruses can cause a wide range of clinical diseases, including herpangina, hand, foot and mouth disease (HFMD), respiratory infections, enteritis, and non-specific febrile illness. In severe cases, enterovirus can lead to aseptic meningitis, encephalitis, myocarditis, and even death.

Enterovirus activity was closely monitored by Centers for Diseases Control of Taiwan (CDC-Taiwan). The most commonly reported serotypes changed each year, as did the ranking of individual serotypes. However, according to the data from CDC-Taiwan during 2001–2008, coxsackievirus A4 (CA4), B3 (CB3) and B4 (CB4) were usually among the five most common enterovirus serotypes in Taiwan [3]. These three coxsackievirus are classified as HEV-A(CA4) and HEV-B(CB3 and CB4), respectively, based on their viral genomic structure. However, detailed clinical features of CA4, CB3 and CB4 infections in children have not been described. In order to better characterize the clinical features of coxsackievirus A4 (CA4), B3 (CB3) and B4 (CB4) infections in children, we conducted this retrospective study.

## Materials and Methods

### Ethical Approval

This study was approved by the Institutional Review Board of Chang Gung Memorial Hospital and the written inform consent was waived since the nature of this study was retrospective chart review.

### Isolation and Identification of Coxsackievirus A4 (CA4), B3 (CB3) and B4 (CB4)

Chang Gung Memorial Hospital (CGMH) is a 3500-bed university-affiliated medical center located in northern Taiwan. Virus isolation from clinical specimens was routinely carried out by the department of laboratory medicine [4]. Specimens were inoculated into human embryonic fibroblast (MRC-5), MDCK, HEp-2 and RD. All cultures were observed daily for cytopathic effects (CPEs). Indirect fluorescent staining with panenteroviral antibody (Chemicon International, Temecula, CA, USA) was performed to identify the enterovirus when CPE involved more than 50% of the cell monolayer. All positive specimens were further confirmed by neutralization with type-specific pools of immune sera. The monoclonal antibodies, including18 serotypes (Poliovirus 1–3; coxsackievirus A9, A16, A24; coxsackievirus B1–6; echovirus 4, 6, 9, 11, 30; EV71), were from a commercial kit (Chemicon International, Temecula, CA, USA), and monoclonal antibodies against coxsackievirus A2, A4, A5, A6 and A10 were provided by CDC–Taiwan since 2006 [5].

### Case Enrollment and Data Collection

From January 2004 to June 2012, a total of 825 isolates of CA4, CB3 and CB4 were identified via an electronic database of virologic laboratory. The virologic laboratory of CGMH was also a contracted laboratory of CDC-Taiwan, which provided the service for viral surveillance in northern part of Taiwan. Patients who didn’t receive treatment at CGMH, or with concomitant viral infection or with a final diagnosis unrelated to enterovirus infection were excluded. Medical records of these cases were reviewed. For outpatients, only demographic data were collected. Inpatients were enrolled for further analysis of clinical manifestations, clinical diagnosis, laboratory data, treatment course and outcomes.

### Definitions

Herpangina was defined as presence of oral ulcers over anterior tonsillar pillars, soft palate, buccal mucosa, or the uvula. Patients with hand, foot and mouth disease (HFMD) had oral ulcers and typical rash over palms, soles, knees, or the buttocks. Diagnosis of tonsillitis/pharyngitis was based on clinical features, such as injected tonsil/throat or presence of exudate. Lower respiratory tract infection (LRTI) included bronchiolitis, bronchopneumonia and pneumonia. Acute gastroenteritis was defined as patients having symptoms of diarrhea and vomiting without other identified viral or bacterial pathogens. Patients younger than three months of age who had fever greater than 38°C but without other symptoms were classified as young infant with fever.

A diagnosis of aseptic meningitis was made when enterovirus was isolated from cerebrospinal fluid (CSF) or pleocytosis in the CSF (leukocyte count in the CSF exceeded 30 cells/mm^3^ in neonates or greater than 5 cells/mm^3^ beyond the neonatal age), with a negative bacterial culture in CSF. Patients who had symptoms and signs of meningeal irritation, such as headache, vomiting, meningeal signs or fever, but no available CSF for analysis or negative results for CSF studies were categorized as meningismus. The diagnosis of encephalitis depended on altered consciousness or focal neurological signs with abnormal findings in neuro-imaging or electroencephalogram (EEG).

Complications included disseminated intravascular coagulopathy, myocarditis, respiratory distress, shock and hepatic necrosis with coagulopathy The diagnosis of disseminated intravascular coagulopathy was confirmed by the scoring system proposed by International Society on Thrombosis and Haemostasis [6]. Myocarditis was defined as an elevation in the cardiac fraction of creatine kinase and ejection fraction less than 50% on echocardiogary or arrhythmia on electrocardiography. Respiratory distress was defined as requiring mechanical ventilation, which included noninvasive modes (e.g., bilevel positive airway pressure, or continuous positive airway pressure) and invasive modes (e.g., pressure-control ventilation, high frequency oscillatory ventilation). Patients who needed inotropic agents due to profound hypotension and accompanied with at least two end-organ injuries were categorized as shock. Hepatic necrosis with coagulopathy was defined as aspartate aminotransferase more than three times the upper limit of normal value plus platelet count less than 10^5^/mm^3^.

### Statistics

The descriptive statistics was performed with SPSS 18.0. The categorical variables were compared by ANOVA and chi-square test. Statistical significance was defined as *p*<0.05.

## Results

**Figure 1:**
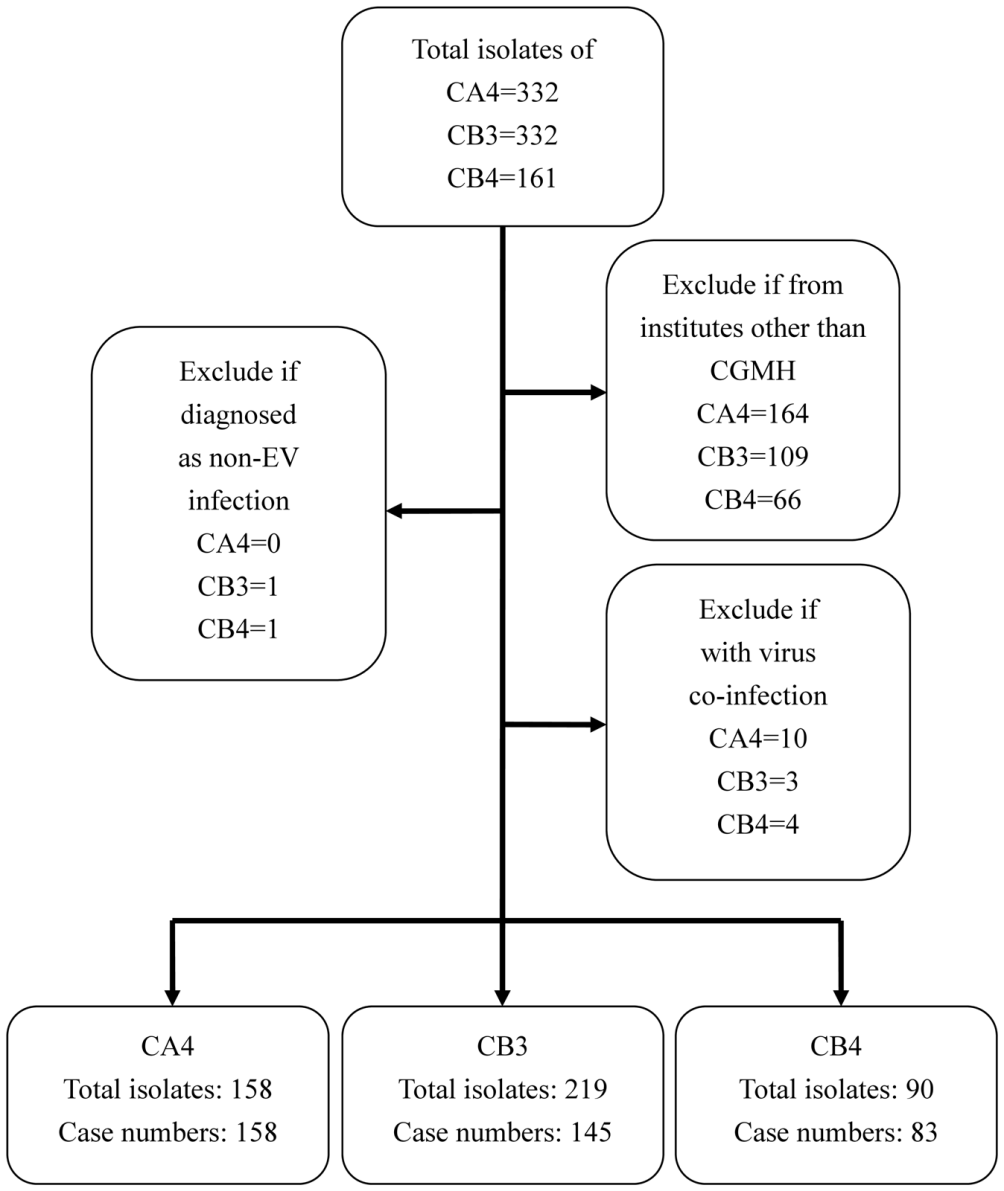
Flow chart of the patients enrolled in this study. CA4, coxsackievirus A4; CB3, coxsackievirus B3; CB4, coxsackievirus B4.

From January 2004 to June 2012, a total of 802 patients less than 18 year of ages with 825 isolates of CA4, CB3 and CB4 which were collected from throat, rectal or CSF were enrolled. 339 patients who didn’t receive treatment at CGMH were excluded. 17 patients were excluded because of other virus co-infection. One patient admitted due to chronic myopathy and the other admitted because of vasculitis were excluded due to no clinical symptoms of enterovirus infection (Fig. 1).

**Figure 2:**
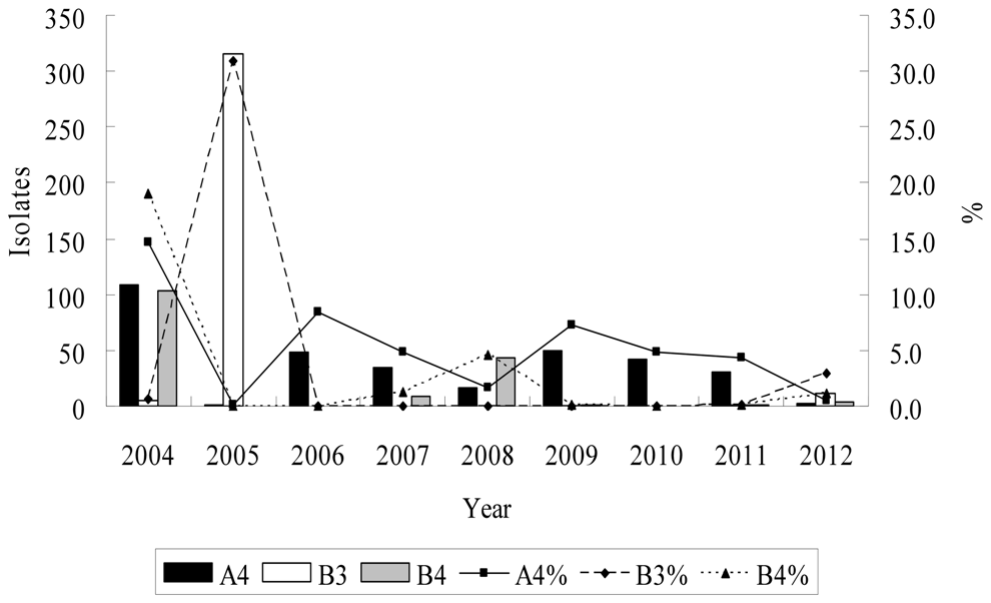
Isolates and percentage of Coxsackie A4, B3 and B4 isolates from January, 2004 to June, 2012 in Chang Gung Memorial Hospital, Taiwan.

**Figure 3:**
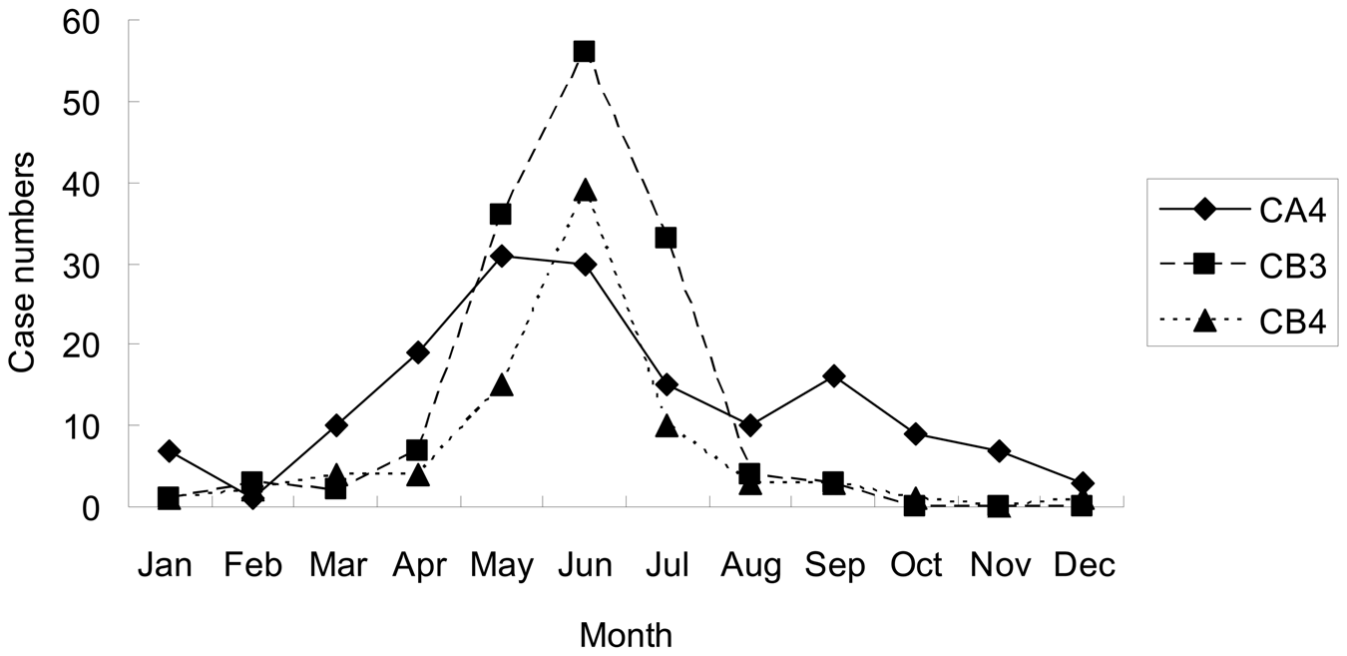
Monthly distribution of coxsackievirus A4 (CA4), B3 (CB3) and B4 (CB4) isolates from January, 2004 to June, 2012 in Chang Gung Memorial Hospital, Taiwan.

A total of 386 children treated at Chang Gung Memorial Hospital, including 296 inpatients (CA4, 103; CB3, 131; CB4, 62) and 90 outpatients (CA4, 55; CB3, 14; CB4, 21), were included in this study. Figure 2 showed the annual distribution and the percentage of CA4, CB3 and CB4 among the total enterovirus isolates which were identified from CGMH during the study period. CA4 and CB4 both had one peak in 2004 and accounted for 14.7% and 19% of the total enterovirus isolates in CGMH, respectively. CB3 constituted 30.9% of the total enterovirus isolates in 2005. On average, from January 2004 to June 2012, CA4 and CB3 each accounted for 5% and CB4 accounted for 2.4% of the overall enterovirus isolates. CA4, CB3 and CB4 were most commonly detected between May and July. The monthly distribution of these three virus during the study period is shown in Figure 3.

The median age of infected children was 25 [10, 49] months (median [IQR]); male to female ratio was 1.3∶1. Children with CB3 infection were youngest (76.6% <3 years of age; 40.7% <3 months of age), and had a highest hospitalization rate (90.3%) and a longest duration of hospitalization (median [IQR], 6 [4], [9] days) (Table 1).

**Table 1:** Demographics of 386 children with Coxsackievirus A4, B3 and B4 infections in Chang Gung Memorial Hospital, January, 2004–June, 2012.

Characteristics	No. (%)				*p* value
	Coxsackie A4	Coxsackie B3	Coxsackie B4	Total	
**Total**	158	145	83	386	
Male gender	81 (51.3)	88 (60.7)	49 (59)	218 (56.5)	.222
Age, months(median [IQR])	30.5 [17.8, 48]	11 [1.2, 33.5]	35 [12, 56]	25 [10,49]	.000
≦3 years	92 (58.2)	111 (76.6)	42 (50.6)	245 (63.5)	.000
≦3 months	1 (0.63)	59 (40.7)	13 (15.7)	73(18.9)	.000
Admission rate (%)	65.2	90.3	74.7	76.7	.000
Specimens					
Throat	158	128	81	367	
Rectum	0	60	8	68	
Cerebrospinal fluid	0	30	0	30	
Others	0	1^a^	1^b^	2	
Multiple sites (≥2 sites)	0	52	7	59	
**Inpatients**	103	131	62	296	
Male gender	54(52.4)	78(59.5)	38(45.8)	170(57.4)	.434
Age, months(median [IQR])	27 [15, 45]	8 [0.9, 32]	24 [7, 53.3]	21 [5,46]	.001
≦3 years	69(67)	105(80.2)	39(62.9)	213(72)	.017
≦3 months	1(1)	59(45)	12(19.3)	72(24.7)	.000
Days of hospital stay (median [IQR])	4 [3], [5]	6 [4], [9]	5 [3], [9]	5 [3], [7]	.000
Underlying diseases	11(10.7)	22(16.8)	12(19.4)	45(15.2)	.257
Prematurity	3	13	6	22	
Allergy	2	4	1	7	
Neurological	3	5	1	9	
Congenital anomaly	1	2	2	5	
Others	0	1	2	3	

a Culture from urine.

b Culture from pleural effusion.

Clinical manifestations and laboratory data of the 296 inpatients are displayed in Table 2 and 3. Of these inpatients, the positive samples for enteroviruses were obtained within three days of hospitalization in all but five patients. The diagnoses of these five patients on admission included herpangina for one, acute tonsillitis for two and lower respiratory tract infection for two. The most common symptoms in the 296 inpatients were fever (97.6%). Children with CA4 infection usually presented with a high-grade fever (75.5%) and those infected with CB3 had a longer duration of fever (55.1% patients had fever greater than 3 days). Patients who were infected by CA4 usually had oral ulcers (80.6%), which is a typical symptom of herpangina, and decreased oral intake (86.4%). Respiratory symptoms were less common in CB3 group. No statistically difference was found for gastrointestinal and neurologic symptoms.

**Table 2:** Clinical manifestations in 296 hospitalized children with coxsackievirus A4, B3 and B4 infections stratified by serotypes.

Symptoms and signs	No. (%)			*p* value
	Coxsackie A4(n = 103)	Coxsackie B3(n = 131)	Coxsackie B4(n = 62)	
**Constitutional**				
Fever^a^	102 (99)	126 (96.2)	61 (98.4)	.330
High fever (>39°C)	74 (75.5)	65 (55.1)	32 (57.1)	.011
Days of fever (median [IQR])	3 [2], [3]	4 [3], [5]	4 [2], [5]	.001
Fever>3 days	23 (23.5)	65 (55.1)	28 (50.9)	.000
Poor oral intake	89 (86.4)	65 (49.6)	34 (54.8)	.000
Poor activity	56 (54.3)	61 (46.6)	26 (41.9)	.261
**Mucocutaneous** b				
Oral ulcer	83 (80.6)	29 (22.1)	25 (40.3)	.000
Skin rash	11 (10.7)	4 (3.1)	3 (4.8)	.039
**Respiratory**				
Cough	51 (49.5)	43 (32.8)	28 (45.1)	.028
Rhinorrhea	38 (36.9)	25 (19.1)	20 (32.2)	.008
**Gastrointestinal**				
Diarrhea	17 (16.5)	12 (9.2)	11 (17.7)	.145
Vomiting	27 (26.2)	27 (20.6)	20 (32.2)	.205
**Neurologic**				
Myoclonic jerk	11 (10.7)	13 (9.9)	8 (12.9)	.823
Headache	2 (1.9)	10 (7.6)	5 (8.1)	.121
Limb weakness	0 (0)	0 (0)	1 (1.6)	.151
Febrile seizure	3 (2.9)	4 (3.1)	3 (4.8)	.773
Afebrile seizure	2 (1.9)	7 (5.3)	1 (1.6)	.247

a Fever was defined as body temperature greater than 38°C.

b Typical mucocutaneous manifestations of enteroviral infections.

**Table 3:** Laboratory findings in 296 hospitalized children with Coxsackievirus A4, B3 and B4 infections stratified by serotypes.

Laboratory data	No. (%)				*p* value
	Coxsackie A4(n = 103)		Coxsackie B3(n = 131)	Coxsackie B4(n = 62)	
**Blood**					
WBC (×1000/µL) on admission	13.9±6.1		11.3±4.0	12.4±5.3	.000
Leukocytosis (peak)^a^	35 (34)		21 (16)	18 (29)	.000
Leukopenia (nadir)^b^	2 (2)		2 (1.5)	1 (1.61)	.745
Hb (g/dL) on admission	11.8±1.2		12.1±2.1	11.8±1.4	.273
Hb<10 g/dL (nadir)	5 (4.9)		29 (22.3)	6 (9.7)	.003
Platelet (1000/µL) on admission	262.1±70.4		299.2±140.5	312.0±127.0	.014
Platelet<150000/µL (nadir)	0 (0)		16 (12.3)	4 (6.5)	.007
CRP (mg/L) on admission	40.5±37.0		20.6±25.9	30.1±53.5	.000
CRP>40 mg/L(peak)	41 (40.6)		22 (16.9)	12 (20)	.000
**Cerebrospinal fluid findings in aseptic meningitis**					
**Case numbers**	0	35		0	
Pleocytosis^c^		31			
WBC (/µL)	–	170±193		–	
Neutrophil (%)	–	17.7±27.7		–	
Lymphocyte (%)	–	18.6±21.4		–	
Protein (mg/dL)	–	93.9±51.8		–	
Sugar (mg/dL)	–	48.5±10.5		–	
Lactate (mg/dL)	–	17.1±4.2		–	
Positive virus culture	–	29		–	

a Leukocytosis was defined as WBC count ≥15000/µL in patients older than 1-month-old and ≥30000/µL in patients young than 1-month-old.

b Leukopenia was defined as WBC count <4500/µL.

c Pleocytosis was defined as WBC count in CSF greater than 30 cells/mm^3^ in neonates or greater than 5 cells/mm^3^ beyond the neonatal age.

Leukocytosis (leukocyte count ≥15000/µL) and higher C-reactive protein (>40 mg/L) were both more frequently seen in children with CA4 infection (34% and 40.6%, respectively). CB3 infection was more likely to develop anemia and thrombocytopenia (22.3% and 12.3%, respectively). 35 patients diagnosed as aseptic meningitis were all infected by CB3. Of them, 31 patients had pleocytosis in CSF and the mean WBC count was 170±193/µL without lymphocyte or netrophil predominance(17.7±27.7% and 18.6±21.4%, respectively). Elevated protein level (mean ± SD, 93.9±51.8 mg/dL) and lower sugar level (mean ± SD, 48.5±10.5 mg/dL) were observed.

Herpangina (74.8%) was the most common diagnosis for children with CA4 infection, aseptic meningitis (26.7%) and young infant with fever (23.7%) for those with CB3 infection, and herpangina (32.3%) and tonsillitis/pharyngitis (27.4%) for children with CB4 infection (Table 4.). Of the 38 patients diagnosed as LRTI, 11 patients were infected by CA4, 17 patients by CB3 and 10 patients by CB4 (Table 5.). Among these patients, most plain chest radiography films were interpreted as having bronchopneumonia (CA4, 90.9%; CB3, 94.1%; CB4, 80%). Positive urine pneumococcus antigen was identified in one patient, 3 patients probably were coinfected with *Mycoplasma pneumoniae* which was supported by either serology or polymerase chain reaction (PCR), and 2 patients had MRSA isolated from sputum cultures.

**Table 4:** Diagnosis, treatment, complications and outcomes of 296 hospitalized children with coxsackievirus A4, B3 and B4 infections stratified by serotypes.

Clinical parameters	No. (%)			*p* value
	Coxsackie A4 (n = 103)	Coxsackie B3 (n = 131)	Coxsackie B4 (n = 62)	
**Diagnosis**				
Herpangina	77 (74.8)	22 (16.8)	20 (32.3)	.000
Hand-foot-mouth disease	5 (4.9)	2 (1.5)	2 (3.2)	.337
Tonsillitis/pharyngitis	14 (13.6)	18 (13.7)	17 (27.4)	.035
LRTI	11 (10.7)	17 (13)	10 (16.1)	.552
Acute gastroenteritis	2 (1.9)	5 (3.8)	11 (17.7)	.000
Young infant with fever	0 (0)	31 (23.7)	9 (14.5)	.000
Aseptic meningitis	0 (0)	35 (26.7)	0 (0)	.000
Meningismus	1 (1)	7 (5.3)	3 (4.8)	.585
Encephalitis	0 (0)	6 (4.6)	0 (0)	.021
Others	8 (7.8)	9 (6.9)	5 (8.1)	.945
More than one diagnosis	14 (13.6)	18 (13.7)	14 (22.6)	
**Treatment**				
Antibiotics	19 (18.4)	77 (58.8)	28 (45.2)	.000
IVIG	2 (1.9)	14 (10.7)	2 (3.2)	.012
**Complications**	1 (1)	12 (9.2)	0 (0)	
DIC	1 (1)	9 (6.9)	0 (0)	.012
Myocarditis	1 (1)	1 (0.8)	0 (0)	.752
Respiratory distress	1 (1)	7 (5.3)	0 (0)	.041
Shock	1 (1)	6 (4.6)	0 (0)	.076
HNC	0 (0)	6 (4.6)	0 (0)	.013
Other	0 (0)	3 (2.3)^a^	0 (0)	.148
**Outcomes**				
Total recovery	102 (99)	121 (92.4)	62 (100)	.006
Long-term sequelae	1 (1)	10 (7.6)	0 (0)	.006
Mortality	1 (1)	1 (0.8)	0 (0)	.752
Requiring ECMO	1 (1)	1 (0.8)	0 (0)	.752

URTI, upper respiratory tract infection; LRTI, lower respiratory tract infection; IVIG, intravenous immunoglobulin; DIC, disseminated intravascular coagulopathy; HNC, hepatic necrosis and coagulopathy; ECMO, extracorporeal membrane oxygenation.

a Two patients complicated with renal failure and one patient had rhabdomyolysis.

**Table 5:** Chest X ray findings and results of etiology survey in hospitalized lower respiratory tract infection (LRTI) patients.

	No. (%)			*p* value
	Coxsackie A4	Coxsackie B3	Coxsackie B4	
**Case numbers of LRTI**	11	17	10	.552
**CXR**				
Increased perihilar infiltrates	10 (90.9)	16 (94.1)	8 (80)	
Patch consolidation	1 (9.1)	1 (5.9)	1 (10)	
Pleural effusion	0 (0)	0 (0)	1 (10)	
**Survey for bacterial co-infections**				
Positive urine pneumococcus antigen	0 (0)	0 (0)	1 (33.3)	
Mycoplasma pneumoniae^a^	0 (0)	2 (33.3)	1 (20)	
MRSA^b^	0 (0)	2 (100)	0 (0)	
RSV antigen (NP)^c^	0 (0)	0 (0)	0 (0)	

a Including mycoplasma serology or mycoplasma DNA PCR.

b MRSA, methicillin-resistant Staphylococcus aureus; confirmed by sputum culture.

c RSV, respiratory syncytial virus; nasopharyngeal aspirate examined by immunofluorescent aassay.

Children with CB3 infection had the highest complication rate (9.2%), with disseminated intravascular coagulopathy being the most common syndrome, followed by respiratory distress, shock and hepatic necrosis with coagulopathy. CB3 infection had a highest rate of receiving antibiotics or IVIG treatment (58.8% and 10.7%, respectively). 10 of 11 children with long-term sequelae were also caused by CB3. Two fatal cases were noted, one due to myocarditis caused by CA4 and t CB3 was detected from the other case which had hepatic necrosis with coagulopathy. The remaining 285 children (96.3%) recovered uneventfully.

In CB4 group, enterovirus was isolated from pleural effusion and throat swab in a case of necrotizing pneumonia. This 4-year-old boy was admitted due to pneumonia complicated with pleural effusion and subsequent chest computed tomography found necrotizing lung tissue during hospitalization. Echo-guided thoracentesis was performed and the results were compatible with an exudate (lactate dehydrogenase 621 IU/mL) according to Light’s criteria. Etiology survey found positive urine pneumococcus antigen, but gram stain, pneumococcus antigen and bacterial culture from pleural effusion were all negative. However, virus culture from pleural effusion and throat specimen which was collected about one week later both yielded CB4. The patient recovered uneventfully without surgical intervention.

In present study, CB3 infection was found to have more severe clinical presentation. We classified patients into CB3 and non-CB3 infection groups for multiple logistic regression analysis. The results showed that patient’s age (adjusted odds ratio, 0.982; 95% confidence interval [CI], 0.971 to 0.992; *P* = .001) and fever duration (adjusted odds ratio, 1.18; 95% confidence interval [CI], 1.06 to 1.314; *P* = .003) were significantly related to CB3 infection. Aseptic meningitis was more frequently diagnosed in CB3 group (adjusted odds ratio, 19.829; 95% confidence interval [CI], 4.876 to 80.649; *P* = .000) as well as complication or mortality (adjusted odds ratio, 13.558; 95% confidence interval [CI], 1.302 to 141.135; *P* = .029). However, length of hospital stay didn’t reach statistic significance (adjusted odds ratio, 1.032; 95% confidence interval [CI], 0.954 to 1.116; *P* = .433).

## Discussion

Results from the present study showed that from January 2004 to June 2012, CA4, CB3 and CB4 each accounted for 2.4–5% of the overall enterovirus isolates. CA4 and CB4 both had a peak in 2004 while CB3 was predominant in 2005. Since 2006, CA4, CB3 and CB4 played less important roles in the enterovirus epidemics. These results were consistent with those from CDC of Taiwan, which revealed that in 2004, the most prevalent serotype was CA4, accounting for 23.8% of total enterovirus isolates, and CB3 constituted 30.6% in 2005 [7]. Whereas, CA4, CB3 and CB4 each constituted 0 to 1.8% of total enterovirus isolates from 1998 to 2007 in Spain, 0.9–2.6% from 2005 to 2006, and 0–3.2% from 2008–2009 in Korea [8]–[10]. From 1970–2005, Khetsuriani N et al. conducted a study based on data from the National Enterovirus Surveillance System in United States, they found CA4, CB3 and CB4 each constituted 0.4%, 3.9% and 4.2% of total enterovirus isolates. CB3 and CB4 were both consistently appeared among the 15 most common enteroviruses [11].

Coxsackieviruses have various serotypes and can cause distinct disease spectrum. In a previous study, Yen FB et al. reported higher hospital admission rate in CB infection than patients with CA infection. CA was commonly related to herpangina while respiratory symptoms were more prominent in CB infection [12]. In another epidemic report in northern Taiwan, Hsu CH et al. not only observed this trend but also identified the association between Human enteroviruses B (including CB1, CB3, CB4, CB5, CA9, Echo3, Echo4, Echo6, Echo25, and Echo30) and aseptic meningitis [13], which was consistent with previous findings [14].

In this study, we found CA4 usually caused herpangina with high grade fever, leukocytosis and higher CRP but rarely caused complications. CB3 tended to infect young children. In the present study, 76.6% patients with CB3 infection were younger than 3 years and half of them were younger than 3 months, which resulted in more frequent usage of empiric antibiotic therapy for these young infants. Aseptic meningitis and young infant with fever were the most common diagnosis and more complications, mostly DIC (9 out of 12 patients with complications developed DIC) were found. In contrast, herpangina and pharyngitis/tonsillitis were the most common diagnosis for CB4 infection and rare complications were noted.

From the enterovirus outbreak in 2005, we learned that CB3 often occurred in infants younger than 3-month-old and caused various manifestations, including fever, meningitis, hepatitis, and sepsis [15]. In this study, we found 35 patients with aseptic meningitis proven by lumbar puncture and all of them were infected by CB3. Most of these patients (91.4%) were younger than 3-month-old (data not shown).

Hepatic necrosis with coagulopathy (HNC) is another important issue in neonatal enteroviral disease. We only collected patients with CA4, CB3 and CB4 infections in this study but already found 6 patients with HNC among 37 neonates and CB3 was detected from all of these cases. In a previous study, Lin et al reported 42 cases had HNC among 146 neonates in northern Taiwan with non-polio entrovirus infections [16]. Another single-center study showed much smaller proportion, from 1999–2006 only one patient with CB3 had developed HNC [12]. Further study is needed to clarify the prevalence of HNC in neonatal enteroviral disease.

Correlations between respiratory tract infections and enterovirus have been reported previously [17], [18]. According to a previous surveillance data of respiratory viral infections among pediatric patients in northern Taiwan, enterovirus is one of the most common viruses identified from pediatric outpatients with acute, febrile URTIs [4]. Tsai et al showed 12.7% children with respiratory tract infections, including upper and lower RTIs, were caused by enterovirus and enterovirus was identified from 20% of viral pneumonia cases [19]. In a study discussing the role of enteroviruses for community acquired pneumonia in adults, Hohenthal et al indicated that enteroviruses could be identified in 5% of the patients [20]. In the present study, 38 patients were diagnosed as LRTIs based on clinical symptoms and chest radiographs. Most children had increased infiltrations on chest radiographs, but patchy opacity and pleural effusion were found in three and one patients, respectively. 25 out of the patients were younger than three years of age. CB4 isolate was identified from pleural effusion from a previously healthy child with necrotizing pneumonia, which was not reported previously, though enteroviruses isolated from lung tissue and bronchoalveolar lavage in immunocompromised patients have been reported [21], [22]. Although concurrent infection couldn’t be excluded, it could be a more direct evidence for enterovius in LRTIs. However, the role of enteroviruses in lower respiratory tract infection is still undetermined, since it is difficult to distinguish colonization from true pathogen without performing simultaneous serologic results. Further studies are needed to address this issue.

There are some limitations in this study. First, this is a retrospective study. Inevitably, there are some missing records or laboratory findings in the medical charts. Second, the presence of clinical features was based on medical records and thus inter-observer’s variation may exist. Third, we generated these data from a single medical center situated in northern Taiwan and the virus cultures were clinical diagnostics requested by treating physicians. Therefore, the results may not be able to apply to other populations. Fourth, because CGMH is a tertiary care center, most severe cases were referred to our hospital and this could be misleading as to the extent of disease spectrums.

In conclusion, we analyzed the clinical features and laboratory findings in children with CA4, CB3 and CB4 infection and found each serotype had distinct clinical presentations. Enteroviruses are common pathogens in children and sometimes can cause morbidity and mortality. A better understanding of the clinical symptoms of enteroviruses infections will be helpful for patient management.
